
JOURNAL INFORMATION
==============================
NLM Title Abbreviation: Biospektrum (Heidelb)
Journal ID: Biospektrum (Heidelb)
NLM Journal ID: 9615685

Biospektrum

ISSN: 0947-0867
EISSN: 1868-6249

ARTICLE INFORMATION
==============================
PMCID: PMC8853039
PMID: 35194331
DOI: 10.1007/s12268-022-1684-y
Article ID: 1684
Article version: 1
Subjects: Wissenschaft Special

Therapeutische Nanobodies gegen SARS-CoV-2

Güttler Thomas 1
Dobbelstein Matthias mdobbel@uni-goettingen.de
2
Görlich Dirk goerlich@mpibpc.mpg.de
1
1 grid.4372.2 0000 0001 2105 1091 Max-Planck-Institut für Multidisziplinäre Naturwissenschaften, Am Fassberg 11, D-37077 Göttingen, Deutschland
2 grid.411984.1 0000 0001 0482 5331 Institut für molekulare Onkologie, Universitätsmedizin Göttingen, Justus-von-Liebig-Weg 11, D-37077 Göttingen, Deutschland
Electronic publication date: 2022 Feb 13
Print publication date: 2022
Volume: 28
Issue: 1
First page: 39
Last page: 42
Copyright: © Die Autoren 2022
License: Dieser Artikel wird unter der Creative Commons Namensnennung 4.0 International Lizenz veröffentlicht, welche die Nutzung, Vervielfältigung, Bearbeitung, Verbreitung und Wiedergabe in jeglichem Medium und Format erlaubt, sofern Sie den/die ursprünglichen Autor(en) und die Quelle ordnungsgemäß nennen, einen Link zur Creative Commons Lizenz beifügen und angeben, ob Änderungen vorgenommen wurden. Die in diesem Artikel enthaltenen Bilder und sonstiges Drittmaterial unterliegen ebenfalls der genannten Creative Commons Lizenz, sofern sich aus der Abbildungslegende nichts anderes ergibt. Sofern das betreffende Material nicht unter der genannten Creative Commons Lizenz steht und die betreffende Handlung nicht nach gesetzlichen Vorschriften erlaubt ist, ist für die oben aufgeführten Weiterverwendungen des Materials die Einwilligung des jeweiligen Rechteinhabers einzuholen. Weitere Details zur Lizenz entnehmen Sie bitte der Lizenzinformation auf http://creativecommons.org/licenses/by/4.0/deed.de.
License URL: https://creativecommons.org/licenses/by/4.0/

issue-copyright-statement: © The Author(s), under exclusive licence to Springer-Verlag GmbH Deutschland, ein Teil von Springer Nature 2022

==============================
Monoclonal immunoglobulins are widely successful as therapeutics and have also been effective in treating COVID-19. However, their production in mammalian cells is expensive and cannot be scaled to meet the demand in a global pandemic. Camelid VHH antibodies (also called nanobodies), however, can be manufactured cost-efficiently in bacteria or yeast. Here we highlight our progress in developing nanobodies that effectively neutralize SARS-CoV-2 and its variants.

■ Sie werden eingesetzt als Immunsuppressiva nach Organtransplantationen, in der Krebstherapie, bei der Behandlung von Autoimmunkrankheiten, Asthma, Allergien, Infektionen und Osteoporose. Auch in der Diagnostik sind sie längst Standard. Die Rede ist von monoklonalen Antikörpern (mAbs). Seit der Entwicklung der Hybridoma-Technologie durch Georges Köhler und César Milstein um das Jahr 1975 [1] und der ersten klinischen Zulassung 1986 haben mAbs die Biomedizin buchstäblich revolutioniert. Sie machen inzwischen knapp ein Fünftel aller neuen Medikamente und etwa die Hälfte der 20 meistverkauften Pharmaka aus [2]. Kein Wunder, denn mAbs verfügen über eine exzellente Zielspezifität und lassen sich genau auf ihre Anwendung zuschneiden. Auch in der COVID-19-Pandemie kommen sie seit November 2020 bei der Prophylaxe und Therapie von Risikopatienten zum Einsatz.

Mit zwei schweren und zwei leichten Polypeptidketten, die über Disulfidbrücken verknüpft sind, ist das typisch Y-förmige Immunglobulin G (IgG) ein relativ großes und flexibles Molekül (**Abb. 1A**). Die Antigenbindestellen (Paratope) werden von den variablen Ig-Domänen der leichten und schweren Kette am Ende der Y-Arme gestellt. Diese passen genau auf die entsprechende Target-Oberfläche (Epitop). Die Fc-Einheit am Fuß des „Y" ist glykosyliert, wodurch IgGs bestimmte Effektorfunktionen aktivieren können. Durch ihre Komplexität müssen mAbs aufwändig aus Säugerzellen gewonnen werden, lange Produktionszeiten und hohe Therapiekosten sind die Folge.

## Kompakte Einzeldomänen-Antikörper im Nanometer-Format

Die Limitierungen von IgGs waren bereits in den 1980er-Jahren abzusehen. Gleich mehrere Laboratorien versuchten daher, kompakte Mini-Antikörper zu entwickeln, die sich kostengünstiger aus Mikroorganismen (wie z. B. *Escherichia coli* oder *Pichia pastoris*) gewinnen lassen. Fab-Fragmente [3] und Einzelketten-Fv-Antikörper [4, 5] sind deutlich kleiner als IgGs, umfassen aber noch mehrere Domänen aus leichter und schwerer Kette (**Abb. 1A**). Kurz nach ihrer Entwicklung wurde entdeckt, dass allein eine isolierte variable Ig-Domäne der schweren Kette eine beträchtliche Antigenaffinität aufweisen kann [6]. Dies ließ auf kostengünstige Einzeldomänen-Antikörper hoffen. Fehlt jedoch die leichte Kette, muss die neu exponierte Oberfläche der schweren Kette so verändert werden, dass die Ig-Domänen nicht aggregieren – kein einfaches Unterfangen, möchte man die ohnehin schwächere Epitopbindung erhalten.

Hier steht
eine Anzeige.
Springer

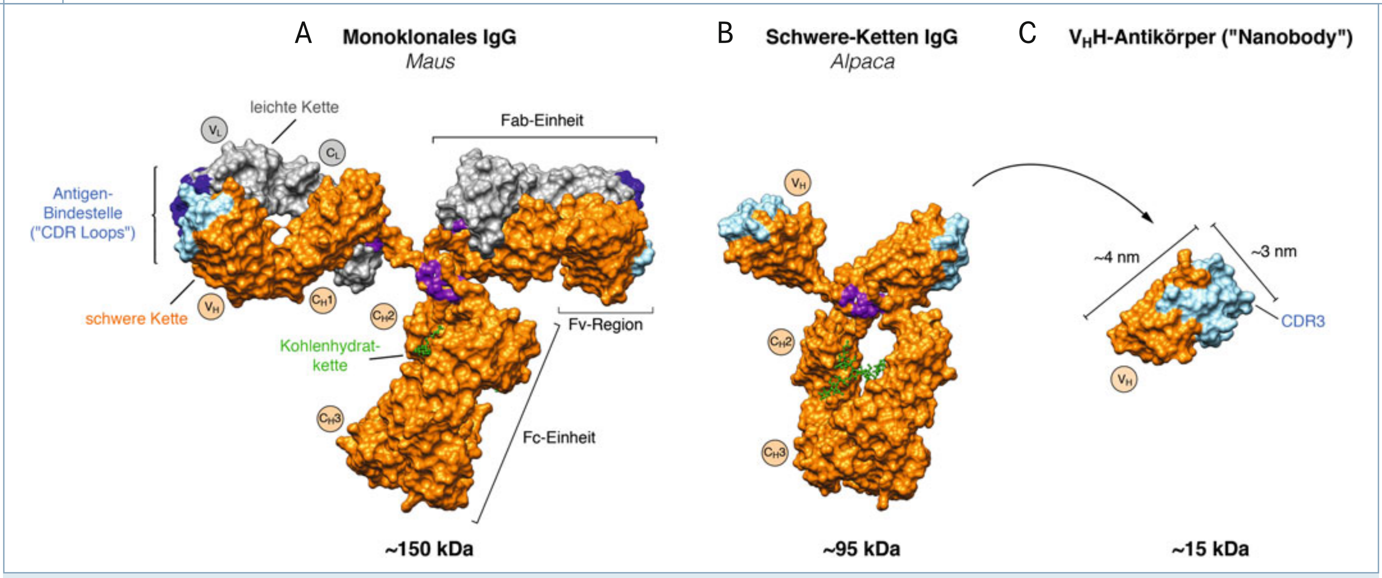

▲ **Abb. 1:** Vergleich von IgGs und Nanobodies. **A,** Struktur von konventionellen IgGs. **B,** Schwere-Ketten IgGs. **C,** Nanobodies. Die konstanten (C) und variablen (V) Ig-Domänen von leichter (L) und schwerer (H) Kette sind markiert. Disulfid-verbrückte Cysteinreste zwischen den Ketten sind violett hervorgehoben. Die Antigenbindestellen sind hellblau (schwere Kette) bzw. dunkelblau (leichte Kette) unterlegt.

Eine Zufallsentdeckung an der Freien Universität in Brüssel brachte in den späten 1980er-Jahren den Durchbruch: Während eines Studentenkurses fielen in Dromedar-Seren Immunglobuline auf, die deutlich kleiner waren als herkömmliche IgGs [7]. Sie bestehen lediglich aus zwei verkürzten schweren Ketten, in denen jeweils eine einzige variable Ig-Domäne das Paratop stellt (**Abb. 1B**). Diese Domänen lassen sich ohne Aggregationsprobleme oder Affinitätsverlust abgetrennt vom Rest der schweren Kette produzieren, als $V_H$-Antikörper (**Abb. 1C**), aufgrund ihrer kompakten Größe auch Nanobodies genannt. Nanobodies lassen sich auch aus anderen Alt- und Neuweltkamelen (wie Alpakas) gewinnen.

## SARS-CoV-2-spezifische Nanobodies

Das Spike-Protein auf der Oberfläche von SARS-CoV-2 ist ein hervorragendes pharmazeutisches Target. Mit dessen Rezeptorbindedomäne (RBD) dockt das Virus an den zellulären Rezeptor ACE2 (*angiotensin-converting enzyme* 2). Daraufhin fusionieren die Membranen von Virus und Zelle, wodurch das Virusgenom in den Wirt eingeschleust wird [8]. Unser Ziel war es, die Interaktion zwischen RBD und ACE2 mit RBD-spezifischen Nanobodies zu blockieren, und SARS-CoV-2 somit zu stoppen, bevor es Zellen infizieren kann [9].

Um solche Nanobodies zu gewinnen, haben wir drei Tiere unserer Alpakaherde mit einer geringen Menge des Spike-Proteins immunisiert. Bereits fünf Wochen später gewannen wir aus etwas Blut periphere B-Lymphozyten – jene Zellen, die auch Spike-Antikörper produzieren. Aus der reverstranskribierten mRNA dieser Zellen ließen sich nun die Nanobody-codierenden Bereiche durch PCR amplifizieren und klonieren. Hier zeigt die Nanobody-Technologie eine ihrer größten Stärken: $V_H$Hs sind viel einfacher zu klonieren als Multidomänen-Antikörper aus mehreren Ketten. In vergleichbar wenigen Schritten ließ sich so das gesamte $V_H$H-Repertoire der Alpakas (~$10^8$ individuelle Sequenzen pro Tier) in einer Nanobody-DNA-Bibliothek (wie man den Satz an Nanobody-kodierenden Plasmiden nennt) spiegeln.

## Die Suche nach der Nadel im Heuhaufen

Die Selektion der antigenspezifischen Nanobodies aus dem Pool aller rekombinanten $V_H$Hs gleicht der Suche einer Nadel im Heuhaufen. Hierbei kommen Bakteriophagen zum Einsatz – Viren, die Bakterien infizieren und deren Biosynthesemaschinerie für ihre Vermehrung kapern. Bei der Konstruktion der Immunbibliothek wird der $V_H$-codierende Bereich mit dem eines Bakteriophagen-Hüllproteins fusioniert. Infiziert man nun Bakterien, die die Bibliothek exprimieren, so präsentiert ein Teil der neu produzierten Phagen einen Nanobody auf der Oberfläche – daher der Name „Phage Display" [10, 11]. Die Plasmide der Bibliothek, die Phagemide, tragen ein Phagen-eigenes Replikationssignal und werden daher in den Phagenpartikeln verpackt. Durch Selektion der Phagen über die präsentierten Nanobodies (mittels Affinitätschromatographie) kommt man so auch der Nanobody-Sequenz auf die Spur. Mit der RBD des Spike-Proteins als Köder fischten wir nun die RBD-bindenden Nanobodies aus der Immunbibliothek und produzierten eine Auswahl dieser in *E. coli*.

## Hochwirksam gegen SARS-CoV-2

Nicht jeder RBD-spezifische Nanobody kann das Virus stoppen. Ob die ausgewählten Nanobodies SARS-CoV-2 ausschalten, ermittelten wir mit einem eigens entwickelten Test, der das Virus sogar im frühen Stadium der Infektion und in einzelnen Zellen detektieren kann [9]. Dafür infizierten wir Zellkulturen mit dem Virus, das zu Beginn des ersten Lockdowns in der Göttinger Universitätsklinik isoliert wurde. Zellen, die infolge der Infektion neue Viruspartikel produzieren, ließen sich mit fluoreszenzmarkierten anti-RBD-Nanobodies per Mikroskop detektieren. Versetzt man das Virus mit einem Nanobody, der die Infektion verhindert, bleibt das Fluoreszenzsignal aus – man spricht dann von Virus-neutralisierenden Nanobodies. 43 von 60 getesteten Nanobodies waren in der Lage, das Virus zu stoppen.

Ein wichtiges Kriterium in der pharmazeutischen Entwicklung ist die minimale Dosis, bei der ein Antikörper wirksam ist. Die verabreichte Menge entscheidet über Nebenwirkungen und nicht zuletzt über Produktionskosten und Marktpreis. Dieser liegt bei dem mAb-Präparat REGEN-COV mit einer Dosis von etwa einem Gramm bei mindestens 2.000 Euro – viel zu hoch für den breiten Einsatz während einer globalen Pandemie. Indem wir die Kandidaten-Nanobodies bei unterschiedlichen Konzentrationen testeten, konnten wir ermitteln, welche Menge für den Neutralisationseffekt ausreicht. Das Ergebnis war erstaunlich: Die potentesten Nanobodies (z. B. Kandidat Re5D06) neutralisierten das Virus bereits bei weniger als einem millionstel Gramm pro Liter. Für einen Patienten könnte also bereits ein Milligramm dieses Nanobodys ausreichen, um die effektive therapeutische Dosis 100fach zu überschreiten [9].

### Mechanismus der antiviralen Aktivität

Mit Immunfluoreszenz-Experimenten erkannten wir bereits früh, dass neutralisierende Nanobodies eines von zwei nicht überlappende RBD-Epitopen (1 oder 2) besetzen, wobei die besten Neutralisierer (wie z. B. Re5D06) Epitop 1 abdecken. Re5D06 gehört gar zu den am stärksten bindenden Nanobodies, die jemals beschrieben wurden. Mittels Röntgenstrukturanalyse ermittelten wir die 3D-Struktur eines Komplexes aus RBD, Re5D06 und einem Epitop-2-Binder (**Abb. 2**). Hier zeigte sich, dass beide Nanobodies die ACE2-Bindung verhindern. Das Paratop von Re5D06 schließt einen auffällig langen CDR3-Loop ein, wodurch der Nanobody formgenau auf Epitop 1 der RBD passt. Solche Bindungsmuster sind eine strukturelle Besonderheit von Nanobodies: CDR3 verleiht ihnen oft eine elongierte, konvexe Gestalt (**Abb. 1C**, [12]). Dadurch passen sie in für IgGs nur schwer zugängliche konkave Oberflächenbereiche und können so auch komplizierte 3D-Epitope lesen und sogar aktive Zentren von Enzymen blockieren [13]. Das Paratop von IgGs ist hingegen meist flach oder konkav, wodurch diese eher lineare Peptide mit hoher Affinität binden [12]. Über Kryo-Elektronenmikroskopie erschlossen wir, dass Re5D06 das Spike-Protein in all dessen Konformationen zu blockieren vermag – es kann sich dem Nanobody praktisch nicht entziehen. Die Röntgenstruktur des freien, nicht RBD-gebundenen Re5D06 zeigte zudem, dass dieser außerordentlich formstabil ist und seine RBD-kompatible Konformation nicht erst allmählich mit der RBD-Bindung einnimmt, sondern diese von Anbeginn aufweist.

### Außergewöhnliche Stabilität

Strukturelle Stabilität von Proteinen geht nicht selten mit Temperaturtoleranz einher. Unsere Favoriten-Nanobodies überstehen sogar eine Temperatur von 95 °C, ohne unwirksam zu werden [9]. Sie zeigen damit eine bisher einzigartige Kombination aus antiviraler Aktivität und Thermostabilität. Letztere gilt sogar als Schlüsselkriterium in der Wirkstoffentwicklung [14]: Zum einen zeigt sie, dass Nanobodies die oft langen Produktions- und Lieferketten unbeschadet durchlaufen können, zum anderen ist sie ein Indikator für lange Wirksamkeit im Körper.

### Schutz vor SARS-CoV-2-Varianten

Seit Herbst 2020 traten immer neue Virusvarianten auf, die das Pandemiegeschehen rasch dominierten. Die nun allgegenwärtigen Delta- und Omikron-Varianten sind ist leichter übertragbar und entziehen sich auch häufiger dem Impfschutz, da die Mutationen vor allem Epitope neutralisierender Antikörper betreffen. Dies führt nicht nur zu Infektionsdurchbrüchen; auch bereits entwickelte therapeutische Antikörper (wie z. B. Bamlanivimab von Eli Lilly) verlieren einen Großteil ihrer Wirkung. Mit der Nanobody-Pipeline lässt sich eine solche „therapeutische Lücke“ zügig schließen: Mittels Phage Display selektierten wir bereits jene Nanobodies aus der Bibliothek unserer besten RBD-Binder, die

Hier steht
eine Anzeige.

Springer

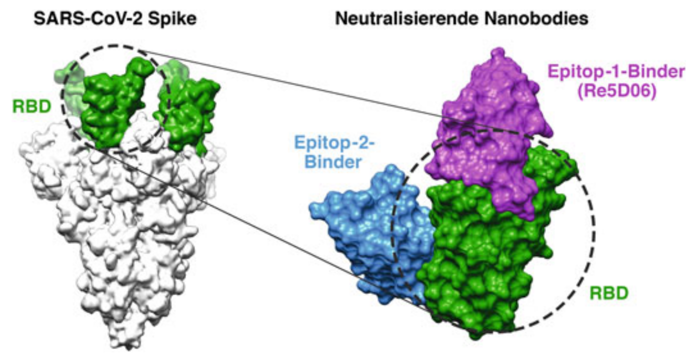

◀ **Abb. 2:** Die Rezeptorbindedomäne (RBD, grün) des SARS-CoV-2 Spike-Proteins (links) wird durch Nanobodies gegen Epitop 1 (magenta) und Epitop 2 (blau) blockiert, wodurch das Virus neutralisiert wird.

trotz der RBD-Mutationen die Alpha-, Beta-, Gamma- und Delta-Varianten des Virus inaktivieren können [9].

Alle RBD-Mutationen vor Omikron der bislang global dominanten Virusvarianten trafen Epitop 1; Epitop 2 blieb bis dahin verschont. Da Epitop-1- und Epitop-2-Binder gleichzeitig dieselbe RBD besetzen können, konnten wir sie zu wirksamen Tandems fusionieren. Diese neutralisieren auch die Virusvarianten Alpha bis Delta und übertrafen sogar die Nanobody-Monomere in ihrer antiviralen Aktivität.

In einer dritten Strategie bündelten wir Epitop-2-Binder zu trimeren Komplexen. So entsprechen sie der Dreier-Symmetrie des Spikes und binden auch die Alpha bis Delta praktisch irreversibel. Nanobodies lassen sich also modular zu effektiven Wirkstoffen kombinieren. Die Nanobody-Pipeline kann man in Echtzeit dem Pandemiegeschehen anpassen. Unsere derzeitige Aufmerksamkeit gilt vor allem der Omikron-Variante.

## Therapeutische Anwendung im Blick

Caplacizumab, ein Thrombozytenaggregationshemmer, ist das bislang einzige zugelassene Nanobody-basierte Präparat [12]. Dass weitere Nanobodies den Weg in die Klinik finden, ist wahrscheinlich. Vor allem dort, wo kompakte Struktur und hohe Stabilität ausschlaggebend sind, werden Nanobodies voraussichtlich konventionellen mAbs überlegen sein. Die intranasale Anwendung oder Inhalation ist für anti-RBD-Nanobodies besonders vielversprechend, denn Nanobodies können leicht in Gewebe eindringen und das Virus bereits am Infektionsort ausbremsen oder sogar stoppen. Erste Ergebnisse aus dem Hamstermodell sind vielversprechend. Falls diese sich bestätigen lassen, könnten klinische Studien bereits im kommenden Jahr beginnen.

## Danksagung

**Korrespondenzadressen:**

Prof. Dr. Dirk Görlich
Max-Planck-Institut für Multidisziplinäre Naturwissenschaften
Am Fassberg 11
D-37077 Göttingen
goerlich@mpibpc.mpg.de

Prof. Dr. Matthias Dobbelstein
Universitätsmedizin Göttingen
Institut für molekulare Onkologie
Justus-von-Liebig-Weg 11
D-37077 Göttingen
mdobbel@uni-goettingen.de

## AUTOREN


Funding note

Open Access funding enabled and organized by Projekt DEAL.

Thomas Güttler 1998–2003 Studium der Biologie und Molekularen Zellbiologie an den Universitäten Jena, Oxford, MS, USA, und Heidelberg. 2004–2010 Promotion am Max-Planck-Institut für biophysikalische Chemie und an der Universität Göttingen. 2010–2019 Postdoktorand an der Harvard Medical School und Boston University, USA. Seit 2019 Wissenschaftler am Max-Planck-Institut für Multidisziplinäre Naturwissenschaften (früher Max-PlanckInstitut für biophysikalische Chemie) in Göttingen.

Matthias Dobbelstein 1986–1992 Medizinstudium an der LMU München mit Promotion am Institut für Biochemie. 1993–1996 Postdoktorand an der Princeton University, USA. 1997–2004 Gruppenleiter am Institut für Virologie der Universität Marburg. 2005–2006 Professor an der Süddänischen Universität Odense. Seit 2006 Professor an der Universitätsmedizin Göttingen.

Dirk Görlich 1985–1989 Biochemiestudium an der Universität Halle. 1990–1993 Promotion am Max-Delbrück-Zentrum/HU Berlin. 1993–1995 Postdoktorand am Wellcome/CRC Institute Cambridge, UK. Forschungsgruppenleiter (1996–2001) und Professor (2001–2006) am Zentrum für Molekularbiologie der Universität Heidelberg (ZMBH). Seit 2005 Direktor am Max-Planck-Institut für Multidisziplinäre Naturwissenschaften (früher Max-Planck-Institut für biophysikalische Chemie) in Göttingen.

Danksagung

Wir danken unseren Arbeitsgruppen am MPI für Multidisziplinäre Naturwissenschaften und an der Universitätsmedizin Göttingen für die hervorragende Teamarbeit an den SARS-CoV-2-Projekten sowie den Facility-Teams am MPI (Tierhaltung, Kristallisation, cryo-EM und Live-Cell Imaging) für die ausgezeichnete Unterstützung. Diese Arbeit wurde von der Max-Planck-Gesellschaft und der Max-Planck-Stiftung gefördert.


Literatur

[1] Köhler G Milstein C Continuous cultures of fused cells secreting antibody of predefined specificity Nature 1975 256 495 497 10.1038/256495a0 1172191
[2] The Antibody Society, www.antibodysociety.org
[3] Better M Chang CP Robinson RR Escherichia coli secretion of an active chimeric antibody fragment Science 1988 240 1041 1043 10.1126/science.3285471 3285471
[4] Skerra A Plückthun A Assembly of a functional immunoglobulin Fv fragment in Escherichia coli Science 1988 240 1038 1041 10.1126/science.3285470 3285470
[5] Huston JS Levinson D Mudgett-Hunter M Protein engineering of antibody binding sites: recovery of specific activity in an anti-digoxin single-chain Fv analogue produced in Escherichia coli Proc Natl Acad Sci USA 1988 85 5879 5883 10.1073/pnas.85.16.5879 3045807 PMC281868
[6] Ward ES Güssow D Griffiths AD Binding activities of a repertoire of single immunoglobulin variable domains secreted from Escherichia coli Nature 1989 341 544 546 10.1038/341544a0 2677748
[7] Hamers-Casterman C Atarhouch T Muyldermans S Naturally occurring antibodies devoid of light chains Nature 1993 363 446 448 10.1038/363446a0 8502296
[8] Jackson CB Farzan M Chen B Mechanisms of SARS-CoV-2 entry into cells Nat Rev Mol Cell Biol 2021 23 3 20 10.1038/s41580-021-00418-x 34611326 PMC8491763
[9] Güttler T Aksu M Dickmanns A Neutralization of SARS-CoV-2 by highly potent, hyperthermostable, and mutation-tolerant nanobodies EMBO J 2021 40 e107985 10.15252/embj.2021107985 34302370 PMC8420576
[10] Smith GP Phage display: simple evolution in a petri dish (Nobel lecture) Angew Chem Int Ed Engl 2019 58 14428 14437 10.1002/anie.201908308 31529666
[11] Winter G Harnessing evolution to make medicines (Nobel lecture) Angew Chem Int Ed Engl 2019 58 14438 14445 10.1002/anie.201909343 31529671
[12] Jovčevska I Muyldermans S The therapeutic potential of nanobodies BioDrugs 2020 34 11 26 10.1007/s40259-019-00392-z 31686399 PMC6985073
[13] Desmyter A Transue TR Ghahroudi MA Crystal structure of a camel single-domain VH antibody fragment in complex with lysozyme Nat Struct Biol 1996 3 803 811 10.1038/nsb0996-803 8784355
[14] Jarasch A Koll H Regula JT Developability assessment during the selection of novel therapeutic antibodies J Pharm Sci 2015 104 1885 1898 10.1002/jps.24430 25821140
